# A87 CHANGES IN CLINICIAN KNOWLEDGE, COMFORT, PREPAREDNESS, AND ATTITUDES ABOUT ALCOHOL USE DISORDER AND CIRRHOSIS AFTER A BRIEF EDUCATIONAL INTERVENTION

**DOI:** 10.1093/jcag/gwab049.086

**Published:** 2022-02-21

**Authors:** E Johnson, S M Ghosh, V J Daniels, P Tandon

**Affiliations:** 1 ^1.^ Medicine, University of Alberta, Edmonton, AB, Canada; 2 ^2.^ University of Alberta, Edmonton, AB, Canada

## Abstract

**Background:**

Alcohol use disorder (AUD) is increasing in prevalence and has a substantial impact on morbidity and mortality in people with cirrhosis. The use of screening, brief intervention and referral to treatment (SBIRT) and relapse prevention medications (e.g. acamprosate) are recommended by recent guidelines. Unfortunately, many clinicians report insufficient training to feel confident using these interventions

**Aims:**

We aimed to compare the effect of a brief educational intervention on AUD knowledge, comfort, attitudes, and preparedness in clinicians who provide care to patients with cirrhosis.

**Methods:**

Clinicians were invited to participate in a 1.5-hour educational session conducted by a hepatologist and addiction medicine specialist. The session included information about SBIRT and pharmacotherapy. Pre-training knowledge, comfort, and practice behaviors were assessed using previously published questions. Baseline attitudes were measured using the Short Alcohol and Alcohol Problems Perception Questionnaire. Participants were invited to repeat the questionnaires immediately post-training and statistical analysis conducted.

**Results:**

Eighty-two clinicians attended the session. Among the 38 attendees who completed both the pre- and post-questionnaires, 34% were GIs/internists, 45% were family medicine physicians, and the remainder (21%) did not specify or were not prescribers. Scores for self-reported intention and preparedness to treat AUD, comfort, and knowledge improved significantly from the pre-training phase. Attitudes also improved from the pre-training phase, with significant improvements in the SAAPPQ subscales of role adequacy (p=0.03) and motivation (p=0.04).

**Conclusions:**

Recognizing the small sample size, a brief educational session demonstrated promising results in the promotion of knowledge, attitudes, preparedness, and comfort for clinicians managing AUD in patients with cirrhosis. Feedback from these sessions will be used to design an accredited educational series for roll-out in 2022.

**Funding Agencies:**

Alberta Innovates Health Solutions

**Table:** Table 1. Paired samples t-test pre and post preparedness, intention and comfort questions

	Pre-test mean (SD)	Post-test mean (SD)	P-value	Effect size
Intention				
To screen every patient for AUD	5.0 (1.2)	5.7 (.74)	<0.001	0.702
Provide medical advice about AUD	5.4 (1.0)	5.8 (0.56)	0.097	0.494
Provide a brief intervention	5.3 (.93)	5.7 (.68)	0.009	0.491
Prescribe anti-relapse medications	4.6 (1.5)	5.2 (1.2)	0.028	0.442
Preparedness				
To screen and diagnose AUD	3.3 (.85)	3.8 (.39)	0.001	.756
To provide a brief intervention	2.8 (.91)	3.6 (.50)	<0.001	1.09
To refer patients for treatment	3.0 (.96)	3.6 (.50)	0.001	.784
Comfort				
Prescribing Acamprosate	2.4 (.82)	3.2 (.75)	<0.001	1.018
Prescribing Baclofen	2.2 (.62)	3.0 (.68)	<0.001	0.279
Prescribing Gabapentin	2.5 (.65)	3.1 (.67)	<0.001	0.909

